# Contrastive Self-Supervised Learning for Stress Detection from ECG Data

**DOI:** 10.3390/bioengineering9080374

**Published:** 2022-08-08

**Authors:** Suha Rabbani, Naimul Khan

**Affiliations:** Department of Electrical and Computer Engineering, Toronto Metropolitan University, 350 Victoria St., Toronto, ON M5B 2K3, Canada

**Keywords:** ECG, contrastive self-supervised learning, affective computing

## Abstract

In recent literature, ECG-based stress assessment has become popular due to its proven correlation to stress and increased accessibility of ECG data through commodity hardware. However, most ECG-based stress assessment models use supervised learning, relying on manually-annotated data. Limited research is done in the area of self-supervised learning (SSL) approaches that leverage unlabelled data and none that utilize contrastive SSL. However, with the dominance of contrastive SSL in domains such as computer vision, it is essential to see if the same excellence in performance can be obtained on an ECG-based stress assessment dataset. In this paper, we propose a contrastive SSL model for stress assessment using ECG signals based on the SimCLR framework. We test our model on two ECG-based stress assessment datasets. We show that our proposed solution results in a 9% improvement in accuracy on the WESAD dataset and 3.7% on the RML dataset when compared with SOTA ECG-based SSL models for stress assessment. The development of more accurate stress assessment models, particularly those that employ non-invasive data such as ECG for assessment, leads to developments in wearable technology and the creation of better health monitoring applications in areas such as stress management and relaxation therapy.

## 1. Introduction

Stress management is essential for maintaining health and wellness. Research shows that prolonged periods of stress contribute to a greater risk for cardiovascular disease, hypertension, decreased immunity, and cancer [1]. Stress is experienced in the event of a perceived threat (stressor), during which our autonomic nervous system (ANS) initiates the ‘fight or flight’ response. In this phase, our body shifts energy from maintaining regulatory processes to more essential functions involved in countering the stressor [2]. As such, stress is a complex phenomenon experienced on multiple levels: behavioral, psychological, and physiological [3].

Physiological signals such as an electrocardiogram (ECG) and galvanic skin response (GSR) signals are greatly influenced in response to a stressor [4]. Studies utilizing ECG signals for stress assessment have recently gained popularity as more evidence suggests its correlation to stress and supports its use in the assessment of psychological health and stress [5]. A thorough literature review of ECG-based emotion assessment in [6] shows an increasing interest in the topic, with 51 papers published from 2004 to 2021.

As data-driven technologies have advanced, the availability of data in such fields has exponentially increased. However, obtaining supervision information (i.e., ground truth labels) associated with this data is a major problem. Usually, these labels are obtained through manual human labeling, which is an expensive and error-prone process due to subjectivity and fatigue [7]. Current ECG-based stress assessment studies mainly utilize supervised learning. However, with the abundance of data available, this poses a limitation to achieving its full potential by not being able to leverage all available data. For ECG-based stress assessment, there have been very limited studies that use self-supervised learning (SSL) to leverage unlabeled data in training, and to our knowledge, none utilize contrastive SSL. A recent study [8] reports improved stress assessment utilizing auxiliary-based SSL. However, with the significant dominance of contrastive SSL in other domains such as computer vision, it is essential to see if the same excellence in performance can be obtained on an ECG-based dataset for stress assessment. In this paper, we propose a contrastive SSL solution based on the SimCLR framework and analyze the performance of it with non-contrastive SSL for ECG-based stress detection. We show that our proposed solution results in a 9% improvement in accuracy on the WESAD dataset and 3.7% on the RML dataset when compared with SOTA ECG-based self-supervised learning models for stress assessment.

## 2. Related Works

### 2.1. Stress Detection from ECG Data

The autonomic nervous system (ANS) regulates the body’s ‘fight or flight’ response in the event of perceived danger or stressor [9]. During this stressed phase, the ANS becomes hyperactive, resulting in physiological and behavioral changes. This makes physiological signals an insightful source of information to assess affective states, i.e., stress. Quantifying the phenomenon of stress still remains a challenging task in research [5]. Recent studies have largely focused on heart rate variability (HRV) to assess stress [5]. HRV is the change in the duration between the R-R intervals of an ECG signal. The significance of HRV and its relation to the ANS make ECG a signal of interest for assessing stress.

In [10], physiological data collected during the real-world driving, the experiment was analyzed by extracting time and frequency domain features. Features extracted from 5 min intervals and 1 s intervals were used to train machine learning models for stress assessment, respectively. The results showed that stress levels were most correlated with skin conductivity and heart rate metrics.

### 2.2. Self-Supervised Learning Applied to ECG Data

In recent years, self-supervised learning has gained popularity because it can leverage more unlabeled data for training. Self-supervised learning is an intermediate concept between supervised and unsupervised learning. It is a two-step framework that generally involves an unsupervised pre-training step followed by the supervised task. First, the model is trained on data using automatically generated labels rather than the ground truth labels. This pre-training step is referred to as the upstream task ($T_{u}$). The following downstream task ($T_{d}$) uses neural network weights from the $T_{u}$ as initialization to improve the training and performance of the actual task at hand. Self-supervised learning generally falls into three categories: auxiliary, generative, and contrastive.

In [8], an auxiliary-based self-supervised learning model is proposed for emotion recognition using ECG data. In the pre-training (upstream) task, a multi-task CNN is used to classify the type of data augmentation applied to unlabelled ECG data. The weights from the upstream CNN are used to initialize the downstream CNN used for emotion recognition. The framework is tested on four publicly available datasets in which it sets state-of-art performance for the classification of arousal, valence, affective states, and stress.

In [11], the authors propose two data augmentation (random baseline drift and random high-frequency interference) techniques unique to 2D images of the ECG data waveform for contrastive self-supervised learning. They compare the performance of their augmentation techniques with popular contrastive SSL architecture, including SimCLR [12], MoCo v2 [13], BYOL [14], SimSiam [15], SwAV [16], and CPC [17] for a medical classification problem. Through their experiments, they found that MoCo v2, along with their proposed augmentations, was the most stable framework.

In [18], a patient-specific contrastive learning algorithm, called CLOCS, is proposed that exploits both temporal and spatial information present within ECG signals. It uses different segments of ECG data from the same patient as positive pairs and uses segments from different patients as negative pairs. They compared with popular contrastive learning methods SimCLR [12] and BYOL [14], and found that CLOCS consistently outperforms on the Chapman and PhysioNet 2020 datasets.

In [19], Mehari et al. investigate the performance of popular contrastive SSL frameworks (SimCLR [12], BYOL [14], SwAV [16], and CPC [17]) on 12-Lead ECG data for classifying diagnostic, form, and rhythm statements. They find CPC to be the best performing framework yielding in a >1% increase in performance. They also notice an improvement in label efficiency and robustness against physiological noise [19].

## 3. Proposed Method

We approach the problem of learning ECG representation for stress detection using contrastive SSL. The application of contrastive SSL has become a popular option in computer vision, natural language processing (NLP) and other fields [20]. Many frameworks have been proposed in the study of contrastive SSL, such as SimCLR, BYOL, and SimSiam [20]. We base our experiments on the SimCLR framework due to its simplicity and dominance in computer vision [12].

Contrastive SSL is a two-step training process that consists of an unsupervised pre-training upstream task ($T_{u}$) followed by a supervised downstream task ($T_{d}$). In the $T_{u}$, a neural network model is trained to learn a high-level representation of ECG data without depending on ground truth labels. Similar to the concept of transfer learning, in the following $T_{d}$ neural network weights from the $T_{u}$ are used as an initialization for training the model to predict stress from ECG data.

### 3.1. Overview of the Upstream Task ($T_{u}$)

**Figure 1:**
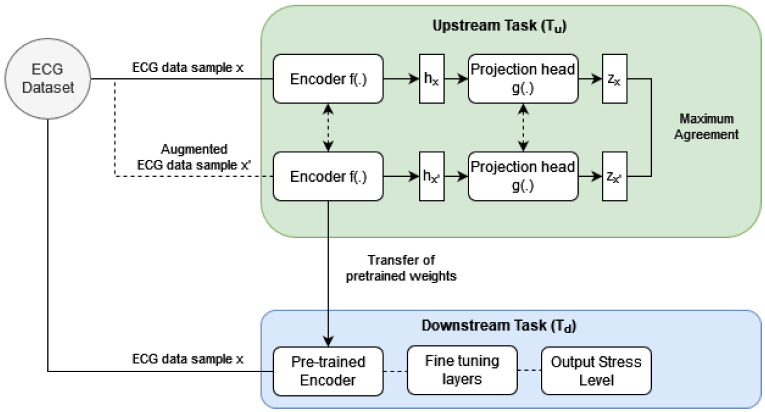
Overview of the contrastive SSL algorithm.

The goal of the $T_{u}$ is to learn a high-level representation of ECG data without depending on ground truth labels. In contrastive SSL, this is done by training the model to minimize contrastive loss. Minimizing contrastive loss trains the upstream model to embed any given data sample and its respective augmented version close together. It also trains the model to embed dissimilar samples away from each other. As seen in Figure 1, data sample *x* and its augmented version $x^{\prime }$ are fed to the siamese twin network in $T_{u}$. The encoder f(.) and projection head g(.) map ECG data into latent space. The latent vectors $Z_{x}$ and $Z_{x^{\prime }}$ correspond to the original ECG sample and its augmented version respectively. An objective function uses the similarity between these vectors to maximize agreement between positive pairs and accordingly updates the weights of the f(.) and g(.).

#### 3.1.1. Upstream Task: Encoder f(.)

Our encoder f(.) is loosely inspired by the 1D CNN presented in [8]. The CNN architecture in [8] has three convolutional blocks followed by a max-pooling layer. These convolutional blocks each consist of 2 convolutional layers with ReLu activation. The filters are increased from 32 to 64 and 128, consecutively in each block, while the kernel size is decreased from 32 to 16 and 8. In our modified architecture, we reduce the number of convolution layers in each block to 1 and add a dropout layer after each max-pooling layer. Following the convolutional blocks is a fully connected layer with 80 hidden units and L2 regularization of size 3 and a flattening layer. The difference in experimental design, i.e., the use of a siamese-twin network has also been a contributing factor that made these changes necessary to avoid pitfalls of this design such as its susceptibility to the vanishing gradient problem [21]. Reducing the number of convolutional layers also led to improving our training efficiency and the dropout layers improved regularization and reduced over-fitting.

### 3.2. Upstream Task: Projection Head g(.)

As demonstrated in the SimCLR study [12], using a non-linear projection head leads to an expressive set of representations in $h_{x}$/$h_{x}^{\prime }$ to be mapped to an invariant set of representations in $z_{x}$/$z_{x}^{\prime }$. This allows for more information on the ECG representation to be maintained in the $h_{x}$ and $h_{x}^{\prime }$ embeddings. Our projection head consists of a fully connected layer with 256 hidden units and softmax activation.

### 3.3. Upstream Task: Data Transformation (Augmentation) Task

The primary transformations explored to create the alter view of *x* are scaling, time warping, permutations, adding Gaussian noise, negation, and horizontal flipping. These are combinations of spatial and temporal transformations that have been successfully used in the upstream task in [8] for ECG emotion recognition. Furthermore, these are fundamental augmentations that are widely used in literature for time series representation learning [22].

### 3.4. Upstream Task: Objective Function

We measure similarity between the $Z_{x}$ and $Z_{x\prime }$ latent vectors using the cosine similarity metric as follows:

(1) $$sim\left(Z_{x},Z_{x^{\prime }}\right)=\frac{{Z_{x}}^{⊤}Z_{x^{\prime }}}{\left\|Z_{x}\|\;\|Z_{x^{\prime }}\right\|}$$

As an objective function, we utilize normalized temperature-scaled cross-entropy (NT-Xent) loss applied to the similarities between a positive pair of latent vectors, i.e.,

(2) $$\ell_{Z_{x},Z_{x^{\prime }}}=-\log\frac{exp(sim\left(Z_{x},Z_{x^{\prime }}\right)/\tau )}{\sum_{\begin{array}{c} y=1\\ y\neq x \end{array}}^{2N}exp\left(sim\left(Z_{x},Z_{y}\right)/\tau \right)}$$

where $Z_{x}$ and $Z_{x\prime }$ are positive pairs, $Z_{x}$ and $Z_{y}$ are negative pairs, and τ is the temperature parameter used for scaling. In each mini-batch, we collect N samples which through NT-Xent loss leads to 2N data points. Each sample N has one positive pair and 2(N-1) negative pairs. Positive pairs are formed between a data sample and its augmented version. Negative pairs are formed between a data sample and every other sample in the batch along with their augmented versions; hence there are 2(N-1) negative pairs. This loss function, along with the cosine similarity metric, has been used in [12]

### 3.5. Downstream Task

The purpose of our $T_{d}$ is to predict stress based on ECG data. It consists of two components; an encoder and fine-tuning layers. The architecture of the encoder in $T_{d}$ is identical to that in the $T_{u}$. It is followed by the addition of fine-tuning layers, including; a flattening layer and a fully connected layer with hidden units equalling a number of classes and softmax activation. After upstream training, the weights of f(.) are used to initialize the stress detection CNN in $T_{d}$.

### 3.6. Dataset

We evaluate our model on the WESAD (Wearable Stress and Affect Detection) [23] and RML (Ryerson Multimedia Laboratory) [24] datasets. The WESAD dataset includes data from 17 participants collected over four tasks, each aimed at inducing a different effect. The affect states studied are neutral, stressed, amused, and meditated. The duration of the collected data is around 33 min for each participant at a sampling rate of 700 Hz. The RML dataset includes ECG data from 12 participants collected during a stress-inducing experience in virtual reality (VR). Participants experienced a dynamic horror roller coaster ride in VR. The stress levels studied are low, medium, and high. The duration of the data collected for each participant is around 3 min at a sampling rate of 256 Hz.

We downsample the WESAD dataset to 256 Hz to match the sampling frequency of the RML dataset [8]. We then remove baseline wander by applying a high-pass Chebyshev filter at a pass-band frequency of 0.8 Hz [8]. Finally, we perform user-specific z-score normalization [8] followed by clipping of the signal where ever saturation of the sensor was observed. Once the data is pre-processed, it is windowed into segments of equal duration. We use a 10 s window for the WESAD dataset. The total samples we obtained for the WESAD dataset are 4569. As the RML dataset is considerably small compared to WESAD, we use a 5 s window to segment the RML ECG signals to increase data points for training. Furthermore, we also apply a data augmentation method, Synthetic Minority Oversampling Technique (SMOTE), to balance out the class distribution in the RML dataset. The total samples we obtain for the RML dataset are 726.

### 3.7. Experimentation

We implement our proposed framework using Keras and train on an Nvidia GeForce RTX 2070. Through our experiments, we aim to evaluate the performance of contrastive SSL vs. non-contrastive SSL for ECG-based stress detection.

**Figure 2:**
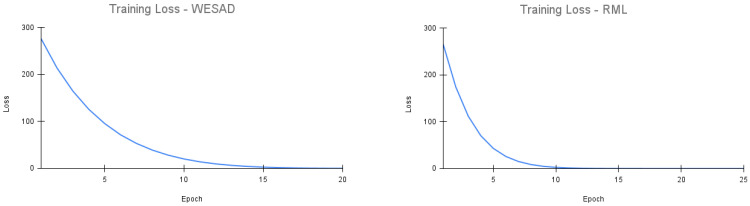
Training loss for $T_{u}$.

On the WESAD dataset, our upstream model is trained using the Adam optimizer with a learning rate of 0.0001 over 20 epochs. On the RML dataset, our upstream model is also trained using the Adam optimizer with a learning rate of 0.0001 over 25 epochs. The batch size used for upstream training on both datasets is 32. Similar to prior works such as [8], the hyperparameters and model architecture for the upstream models were tuned based on the training set. The variety and combination of tuning parameters tested were based on empirical reasoning and a series of trial and error experiments. In particular, the learning rate, batch size, and training epochs were configured based on decreasing training loss. This is because the metric performance, i.e., accuracy, F1 score, etc., of the upstream model serves no purpose in stress detection. The purpose of the upstream model is to learn a high-level representation of the dataset is achieved based on the training performance. Figure 2 depicts the training loss of the upstream model for the WESAD and RML datasets, respectively. We see that the loss stabilizes well by the end of training. Furthermore, the upstream model was trained on the entire WESAD and RML dataset, respectively, for the WESAD and RML experiments. The use of the entire dataset for upstream training is seen in prior studies for self-supervised learning, for example, [8]. As the upstream model does not use any actual ground truth labels but rather trains on pseudo or automatically generated labels, bias due to repetition of data is not a major concern.

The downstream model is trained using the Adam optimizer with a learning rate of 0.001. The $T_{d}$ uses a batch size of 128 and is trained over 250 epochs for the WESAD and 150 epochs for the RML dataset. The hyperparameters for the downstream model are also fine-tuned based on decreasing training loss. Ideally, a validation set to tune the hyperparameters would have been a better experimental setup. However, with the RML dataset being limited in size, we chose to perform hyperparameter tuning based on the training performance. After hyperparameter tuning, we perform our downstream experiments in 10-folds, with random shuffling of data in each fold. In each fold, the data is split into a given train and test ratio. The downstream model is initialized using the weights of the upstream encoder and trained using the train set. The model’s performance is then tested on the test set.

We perform a detailed ablation study to understand the impact of single augmentations versus using a composition of augmentations in $T_{u}$. Similar to the approach used in the SimCLR [12] experiments, we analyze the downstream performance based on varying augmentation combinations used in $T_{u}$. Furthermore, we also study the impact of pre-training on varying ratios of the test to train data used in the $T_{d}$.

We compare our contrastive SSL model’s performance to that of three benchmarks: non-contrastive SSL, fully-supervised learning, and classical machine learning with extracted HRV features. The non-contrastive SSL benchmark used in our experiments is the one presented in [8]. This is an auxiliary-based self-supervised learning model, which to our knowledge is the only other self-supervised learning approach used for stress prediction from ECG data. Comparing this benchmark allows us to assess the performance of contrastive vs. non-contrastive SSL for ECG-based stress detection. The fully supervised benchmark is identical to the 1D CNN described for the $T_{d}$. The only difference it has to our contrastive SSL framework is the lack of upstream pre-training. Essentially, this benchmark helps us assess the improvement in performance we see with upstream pertaining. Besides assessing stress from ECG data, the use of HRV features are widely used in literature and is considered the gold standard for stress assessment [5,25]. We train a support vector machine (SVM) on HRV features calculated from ECG data. We extracted the following HRV features, following the work [26]: Heart Rate (HR), Root Mean Square of Successive Differences (RMSSD), Average Value of N-N Intervals (AVNN), Standard Deviation of N-N Intervals (SDNN), pNN50, Very Low Frequency (VLF), Low Frequency (LF), High Frequency (HF), and Total Power Spectrum (TP). These experiments were also performed in 10 folds with a random shuffling of data in each fold.

## 4. Results

In this section, we present the results of our contrastive SSL model on the WESAD and RML datasets.

### 4.1. Results on WESAD Dataset

**Figure 3:**
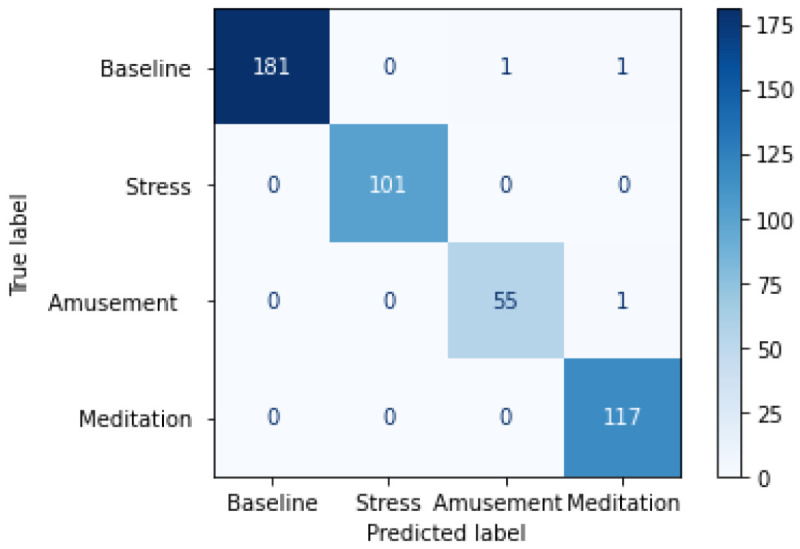
Confusion matrix for contrastive SSL model on the WESAD dataset.

Our proposed contrastive SSL model uses time-warping followed by scaling augmentations applied sequentially for upstream training. The choice of this configuration is explained further in later sections. It is pretrained on the entire WESAD dataset in the $T_{u}$. The weights are then transferred to the $T_{d}$ where the model is fine-tuned for the 4-class affect detection problem. As seen in Table 1 we obtain an accuracy of 94.04% using a test-train split of 10–90. Furthermore, the confusion matrix in Figure 3 displays how accurately it is able to predict each class.

#### 4.1.1. WESAD: Comparison of Results with State-of-Art

**Figure 4:**
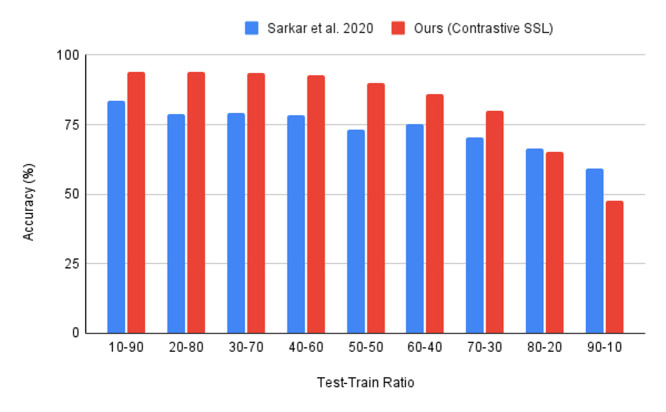
Contrastive SSL (proposed) vs. non-contrastive SSL [8] for varying test-train splits on the WESAD dataset.

In [8], a non-contrastive SSL model is proposed for emotion detection using ECG data. This model is pretrained on four publicly available affect-based datasets; AMIGOS, DREAMER, WESAD, and SWELL. When pretrained on the four datasets, it achieves an accuracy of 95% on the WESAD dataset. However, if only pretrained on the WESAD dataset, it achieves an accuracy of 86–87%. We recreate the experiments of [8] and compare it with our model for the varying test–train ratios, as shown in Figure 4.

#### 4.1.2. WESAD: Ablation Study

Table 2 displays the results obtained from the test-train ratio ablation study. In this experiment, the impact of pre-training with the primary augmentation was studied independently while varying the test-train ratio of the downstream task.

**Figure 5:**
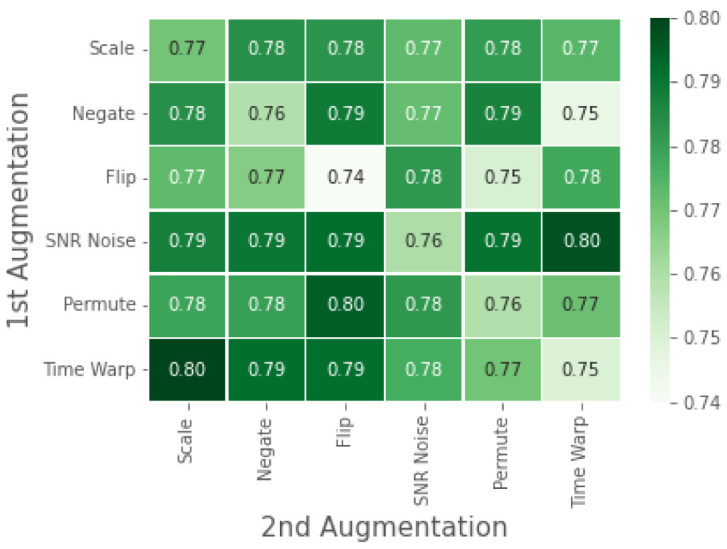
Contrastive SSL evaluation of WESAD under single or composition of data augmentations. Diagonal values correspond to single augmentations, while off-diagonals correspond to the composition of two augmentations applied sequentially.

In Figure 5, we present the accuracy of our downstream model with varying configurations of augmentations used in the $T_{u}$. We chose to perform the augmentation ablation study on the 70–30 split only, as it resulted in the maximum improvement from pre-training. Previous works such as [12,27] have performed this ablation study using linear evaluation of the downstream task using learned ECG representation from the upstream task. The left to right diagonal axis shows downstream results obtained from pre-training using a single augmentation, while the rest of the results correspond to pre-training with a composition of augmentations.

### 4.2. Results on RML Dataset

**Figure 6:**
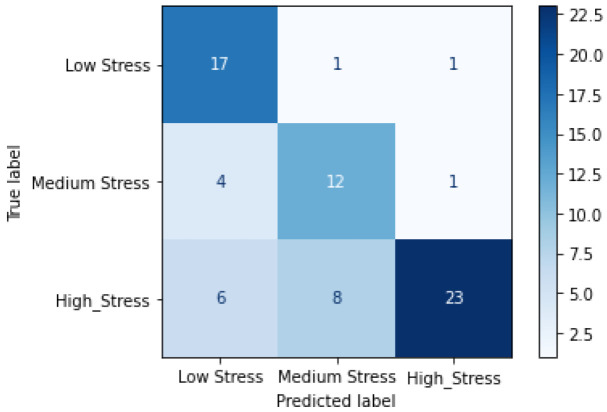
Confusion matrix for contrastive SSL model on the RML dataset.

For the RML dataset, our proposed contrastive SSL model only uses the time-warping augmentation in the $T_{u}$. The choice of this configuration and exception to using the composition of augmentations are explained further in later sections. It is pretrained on the entire RML dataset in the $T_{u}$. The weights are then transferred to the $T_{d}$ where the model is fine-tuned for the three stress-level detection problems. We obtain an accuracy of 73.8% using a test-train split of 10–90. Furthermore, the confusion matrix in Figure 6 displays our models’ accuracy for predicting each class.

**Figure 7:**
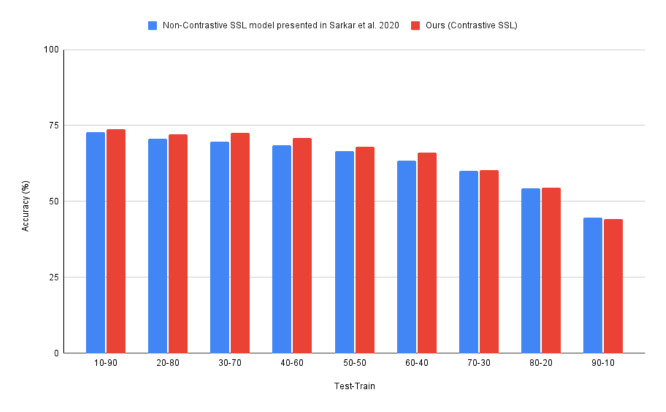
Contrastive SSL (proposed) vs. non-contrastive SSL [8] for varying test–train splits on the RML dataset.

We further evaluate our proposed model by comparing it to the non-contrastive SSL method [8]. Table 3 shows that our contrastive SSL model has a 3.7% improvement when compared to non-contrastive SSL. In Figure 7, we see the performance of our model for the stress level detection problem for varying test-train ratios.

### 4.3. RML: Ablation Study

**Figure 8:**
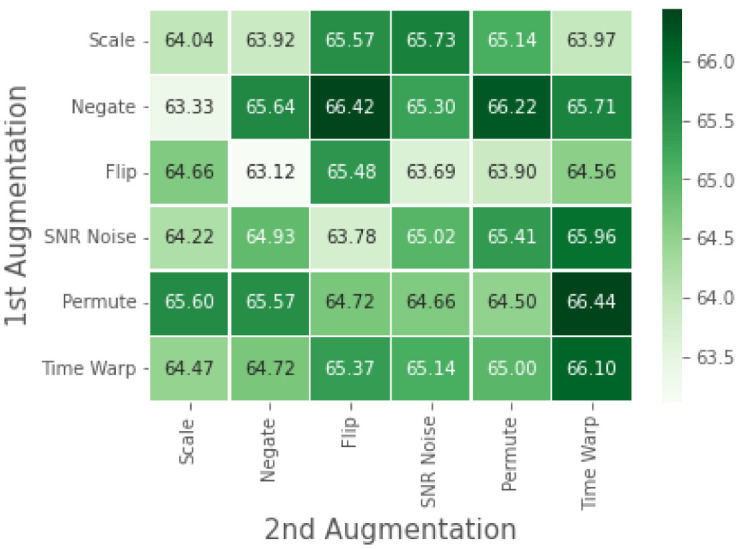
Contrastive SSL evaluation of RML under single or composition of data augmentations. Diagonal values correspond to single augmentations, while off-diagonals correspond to the composition of two augmentations applied sequentially.

We analyze our contrastive SSL model on the RML dataset in a similar experimental setup used for the ablation studies performed on WESAD. Table 4 displays the results obtained from the test–train ratio ablation study on the RML dataset. While Figure 8 presents results for the augmentation ablation study.

### 4.4. Class Imbalance in RML Dataset—Influence of SMOTE

Prior to performing any experiments, the class imbalance found in the RML dataset was addressed using oversampling of the minority classes. As shown in Table 5, the ratio of the classes is 121:44:24. To combat the imbalance, we perform Synthetic Minority Oversampling Technique (SMOTE) to match the number of data samples in the minority classes to the number of samples in the majority class. We analyze the impact of SMOTE on the RML dataset by comparing the performance of the fully supervised benchmark with and without SMOTE. As seen in Table 6 we notice a difference of 27.6% in accuracy after applying SMOTE.

## 5. Discussion

This study proposes a solution based on contrastive SSL for ECG-based stress detection. We investigated the performance of our contrastive SSL model versus non-contrastive SSL, fully-supervised learning, and a machine learning approach based on extracted HRV features. The experiments were carried out on the WESAD and RML datasets.

### 5.1. Analysis of Results on the WESAD Dataset

As seen in Table 1 our results on the WESAD dataset consistently demonstrate that upstream pre-training significantly helps the performance of our downstream stress detection task when compared with the given benchmarks. Furthermore, the confusion matrix in Figure 3 shows that our model can successfully classify the baseline and stress tasks with no false negatives. It also classifies the amusement and meditation tasks well, with only three instances of false prediction.

We compare our results with the auxiliary self-supervised learning approach for ECG-based stress detection proposed in [8]. The pre-training upstream task of this method employs a multi-task CNN to predict the type of augmentation applied to a given data sample. After pre-training, the weights of the CNN are used to aid in training the stress prediction CNN in the downstream task. In comparison, our upstream task uses a Siamese twin network to compare the similarity between two data samples. After pre-training, the weights from the base CNN (encoder f(.)) are used to aid downstream training of the stress prediction CNN. The major differences between [8] and our model lies within the upstream task and structure of the base CNN (encoder). The upstream training in [8] is over 100 epochs while ours is over 20 epochs. Furthermore, as mentioned in Section 3.1.1, the architecture of our base CNN is much simpler, being nearly half the size of the one used in [8].

We find that our solution provides a more effective and superior performance when compared to the non-contrastive SSL approach in [8]. Our contrastive SSL model outperforms the non-contrastive SSL approach by 9% in a one-to-one comparison with [8]’s SSL model, having only been pre-trained on WESAD. Furthermore, our approach is comparable to the performance of [8] having been pretrained on four ECG datasets, achieving a near performance (<1% difference) with simply having been pretrained on WESAD, employing a shallower CNN model and a simpler pretext task. As seen in Figure 4, our model, having been trained on only 30% of the dataset (30% training set), matches the performance of non-contrastive SSL, having been trained on 80% of the dataset. This goes to show the full potential of our model is seen for larger tests and smaller train sets, a likely scenario in real-world settings.

The test–train ablation study performed on the WESAD shows that improvement in $T_{d}$ peaks at the 70–30 ratio and starts to drop onward as the amount of unlabelled data used for training is increased. An exception of this is seen with the horizontal flip augmentation, which peaks at the 80–20 ratio. The performance between the 10–90 ratio up to the 50–50 ratio is minimal compared to that seen beyond this point. This suggests that up to this point, training data suffices ECG representation learning. However, beyond this point, the pretrained weights significantly aid in the learning up to the 80–20 ratio. This trend in the performance of $T_{d}$ is observed with almost all varying augmentation explored for upstream training.

Our proposed contrastive SSL model uses time-warping followed by scaling augmentations applied sequentially for upstream training on WESAD. This configuration of augmentations was chosen based on the ablation study presented in Figure 5. As seen in Figure 5, besides the scale and negate augmentations, the rest of the single augmentation tasks performed poorly compared to when used in the composition of another augmentation. Time warping applied as a first augmentation is observed to be effective, while the flip and scale method for second augmentations has superior performance. While comparing all combinations, time warping followed by scaling has the best performance. This makes intuitive sense; while the time warping augmentation provides temporal information, the scaling augmentation provides spatial information.

## 5.2. Analysis of Results on the RML Dataset

Our model’s performance on the RML dataset confirms our hypothesis that contrastive SSL is superior to non-contrastive SSL for learning ECG representation for stress assessment. Although the improvements are not as consistent as those observed in WESAD; our model outperforms all of the studied benchmarks as seen in Table 3. Further, the confusion matrix in Figure 6 shows that our model can classify the three levels of stress well. It classifies the high-stress levels the best with only two instances of false positives. However, there is some area for improvement in terms of the model’s false negative results, especially with the medium stress class. Some of the inconsistencies in performance on the RML dataset could be attributed to the limited size of the dataset and the use of a 5 s window rather than the 10 s window used for the WESAD dataset. However, even for the smaller RML dataset, the performance of our method is mostly better when compared to the benchmarks.

The test-train ablation study shows that improvement in accuracy from using pretrained weights is noticed until the 60–40 split. Beyond this ratio, the effect of pre-training drops. Similar results were observed in the WESAD dataset, where the improvement in accuracy drops beyond the 70–30 test-train split. While improvement peaked at 70–30 split for the WESAD dataset, the improvements in the RML data are distributed, with most of them occurring up to 60–40 split.

The augmentation ablation study seen in Figure 8 shows that time warping offers greater ECG representation learning when applied as a single or second augmentation. However, the composition of augmentations, in this case, does not result in significant improvement; thus, the use of single time-warping augmentation is a more efficient option. Since the RML dataset is limited in size, the composition of augmentation is too difficult of a task to learn with limited datapoints.

## 5.3. Real World Applications

On the WESAD dataset, our model, having been trained on only 30% of labeled samples, matches the performance of non-contrastive SSL, having been trained on 80% of labeled samples, showing the potential of contrastive SSL for ECG representation learning. The ability to accurately assess stress from minimally labeled data paves the way for advancements in stress management and relaxation therapy applications. Stress is a complex phenomenon that affects each individual differently. As our model can be fine-tuned on limited data while still producing accurate results, it can be used to create personalized stress inference. The upstream model, fine-tuned on a small amount of patient-specific labeled data, can provide personalized stress assessment for each user. Furthermore, stress inference based on non-invasive data such as ECG paves the way for developing more user-friendly applications that can run on commodity hardware.

## 6. Conclusions

As technology advances, its applications are found in many aspects of our everyday lives, such as education, wellness, and communication. This increased presence of technology has led to research in the area of affective computing to create a more engaging user experience. Affective computing is the study of adding emotional intelligence to an application. It enables a machine to understand and respond to the users’ affect (mood or feelings), such as stress [28]. Most of the current studies utilize supervised learning for stress detection with ECG data, whether it be using classical machine learning or deep neural networks. In the area of stress assessment from ECG data, there have been very limited studies that use self-supervised learning to leverage unlabelled data in training, and none that use contrastive self-supervised learning.

In this study, we propose an ECG-based stress assessment solution that leverages unlabeled data in training using contrastive SSL. We compare our results with existing works, which are based on non-contrastive SSL, fully supervised learning, and machine learning using HRV features. The performance of our contrastive model confirms our hypothesis of contrastive SSL being a superior solution for ECG-based stress detection. Our proposed algorithm results in a noticeable improvement in accuracy when compared with the given benchmarks.

The development of more accurate stress assessment models that can leverage unlabelled ECG data for training opens up a plethora of health and wellness applications. In stress management and relaxation training therapies, the application of affective computing have proven to be very successful. Affective state measured through physiological signals such as ECG can provide the user with insight (biofeedback) about their effect. This information can then be used to train the individual to alter their physiological activity to reach a more rested or desired affective state [29].

While stress classification based on a 5 or 10 s window offers, a good indication of one’s changing affect/mood. A more seamless solution could be based on a regression approach rather than classification. In the future, we plan on assessing stress on a continuous scale rather than discrete classifications to provide a more variant scale of stress levels. The use of such models will be ideal in biofeedback-based applications for relaxation therapy.

We chose to implement SimCLR for ECG-based stress detection because it is the SOTA contrastive SSL framework on the ImageNet dataset [30]. It would be interesting, however, to compare the performance of other popular contrastive SSL methods, as done in [19].

## Figures and Tables

**Table 1:** Our results compared with supervised baseline and prior works on the WESAD dataset. All of the experiments presented in this table use a 10–90 test–train split.

Method	Accuracy ± Standard Deviation
Machine Learning - SVM	46.43 ± 1.53%
Fully Supervised	93.02 ± 3.03 %
Non-contrastive SSL [8]	87% ^1^
Ours (Contrastive SSL)	94.09 ± 1.30 %

^1^ Value taken from [8]—standard deviation not provided.

**Table 2:** Performance of the downstream task $T_{d}$ with varying test–train ratios explored for each primary augmentation separately.

Signal Transformation Task							
**Test-Train Ratio**	**No Augmentation**	**Scale**	**Negate**	**H-Flip**	**SNR Noise**	**Time Warp**	**Permute**
10–90	93.0	92.0	93.5	93.4	94.6	93.6	94.2
20–80	92.8	93.0	92.7	92.4	92.4	93.6	92.5
30–70	91.9	90.6	91.8	90.8	92.0	88.7	90.1
40–60	89.9	91.0	88.9	91.0	89.7	89.8	91.7
50–50	85.9	84.9	86.2	88.5	87.4	88.6	87.7
60–40	80.6	82.3	82.1	83.9	83.8	84.0	85.4
**70–30**	67.1	77.01	75.9	74.0	76.0	75.0	75.9
80–20	55.8	59.6	58.3	62.2	60.8	62.6	60.9
90–10	43.4	48.4	48.1	48.6	45.9	46.4	45.5

**Table 3:** Our results compared with supervised baseline and prior works on the RML dataset. All of the experiments presented in this table use a 10–90 test–train split.

Method	Mean Accuracy ± Standard Deviation
Machine Learning - SVM	59.5 ± 4.97%
Fully-Supervised	72.9 ± 4.3%
Non-contrastive SSL [8]	70.1 ± 3.4 %
Contrastive SSL (ours)	73.8 ± 8.7%

**Table 4:** Performance of the $T_{d}$ with varying test-train ratios was explored for each primary augmentation separately. This table presents the results obtained from the RML dataset.

Signal Transformation							
**Test-Train Ratio**	**No Augmentation**	**Scale**	**Negate**	**H-Flip**	**SNR Noise**	**Time Warp**	**Permute**
10–90	72.9	76.6	74.1	73.0	74.7	73.8	75.2
20–80	70.8	72.9	74.8	72.6	72.3	72.1	72.7
30–70	69.6	70.9	73.8	72.7	72.7	72.6	72.9
40–60	68.6	70.7	69.7	71.4	71.3	70.8	72.1
50–50	66.7	68.3	69.6	68.9	69.7	68.0	67.5
**60–40**	63.3	64.0	65.6	65.6	65.0	66.1	64.5
70–30	60.1	61.2	59.7	60.6	61.0	60.3	60.9
80–20	54.3	54.5	53.6	55.5	53.9	54.5	54.9
90–10	44.8	44.9	43.9	43.7	44.0	44.1	44.6

**Table 5:** Displays the class imbalance found in the RML dataset.

Class	Number of Samples in Dataset
Low Stress	242
Medium Stress	88
High Stress	48

**Table 6:** The comparison of the performance of the fully supervised learning benchmark using RML dataset having been over-sampled with SMOTE.

Method	Mean Accuracy ± Standard Deviation
Fully Supervised Benchmark without SMOTE	45.3 ± 4.04%
Fully Supervised Benchmark with SMOTE	72.9 ± 4.3%

## Data Availability

In this paper, we present results obtained from WESAD and RML datasets. The WESAD dataset was accessed on 1 October 2019 via: https://uni-siegen.sciebo.de/s/HGdUkoNlW1Ub0Gx/download. The RML dataset is not publicly available.
